# Force-Induced Changes of PilY1 Drive Surface Sensing by Pseudomonas aeruginosa

**DOI:** 10.1128/mbio.03754-21

**Published:** 2022-02-01

**Authors:** Shanice S. Webster, Marion Mathelié-Guinlet, Andreia F. Verissimo, Daniel Schultz, Albertus Viljoen, Calvin K. Lee, William C. Schmidt, Gerard C. L. Wong, Yves F. Dufrêne, George A. O’Toole

**Affiliations:** a Department of Microbiology and Immunology, Geisel School of Medicine at Dartmouth, Hanover, New Hampshire, USA; b Louvain Institute of Biomolecular Science and Technology, Université Catholique de Louvain, Louvain-la-Neuve, Belgium; c Institute for Biomolecular Targeting (bioMT), Geisel School of Medicine at Dartmouth, Hanover, New Hampshire, USA; d Department of Bioengineering, University of California, Los Angeles, California, USA; e Department of Chemistry and Biochemistry, University of California, Los Angeles, California, USA; f California NanoSystems Institute, University of California, Los Angeles, California, USA; Nanyang Technological University

**Keywords:** type 4 pili, force, PilY1, von Willebrand A domain, surface sensing, c-di-GMP

## Abstract

During biofilm formation, the opportunistic pathogen Pseudomonas aeruginosa uses its type IV pili (TFP) to sense a surface, eliciting increased second-messenger production and regulating target pathways required to adapt to a surface lifestyle. The mechanisms whereby TFP detect surface contact are still poorly understood, although mechanosensing is often invoked, with few data supporting this claim. Using a combination of molecular genetics and single-cell analysis, with biophysical, biochemical, and genomics techniques, we show that force-induced changes mediated by the von Willebrand A (vWA) domain-containing, TFP tip-associated protein PilY1 are required for surface sensing. Atomic force microscopy shows that TFP/PilY1 can undergo force-induced, sustained conformational changes akin to those observed for mechanosensitive proteins like titin. We show that mutation of a single cysteine residue in the vWA domain of PilY1 results in modestly lower surface adhesion forces, reduced sustained conformational changes, and increased nanospring-like properties, as well as reduced c-di-GMP signaling and biofilm formation. Mutating this cysteine has allowed us to genetically separate a role for TFP in twitching motility from surface-sensing signaling. The conservation of this Cys residue in all P. aeruginosa PA14 strains and its absence in the ∼720 sequenced strains of P. aeruginosa PAO1 may contribute to explaining the observed differences in surface colonization strategies observed for PA14 versus PAO1.

## INTRODUCTION

Pseudomonas aeruginosa is a ubiquitously distributed opportunistic pathogen that encounters mechanical forces during surface sensing, a crucial first step for biofilm formation. Their motility appendages, type IV pili (TFP), are integral to surface sensing and is thought to transduce a force-induced signal to the cell interior by detecting the resistance to retraction when cells are surface engaged (1), activating the production of cAMP and c-di-GMP and regulating target genes that control biofilm formation (2–4). While the importance of the TFP and its tip-associated protein, PilY1, in surface sensing has been proposed (4, 5), direct evidence of how the TFP and PilY1 sense the surface is lacking. Indeed, much of the supporting evidence of a role for this appendage as a key surface sensor is deductive or, alternatively, relies on biological responses or phenotypic changes that are observed during the switch from planktonic to surface-attached growth. In this study, we thus take a multidisciplinary approach to investigate the mechanism whereby the TFP via the tip-associated protein, PilY1, is directly involved in surface sensing.

**FIG 1:**
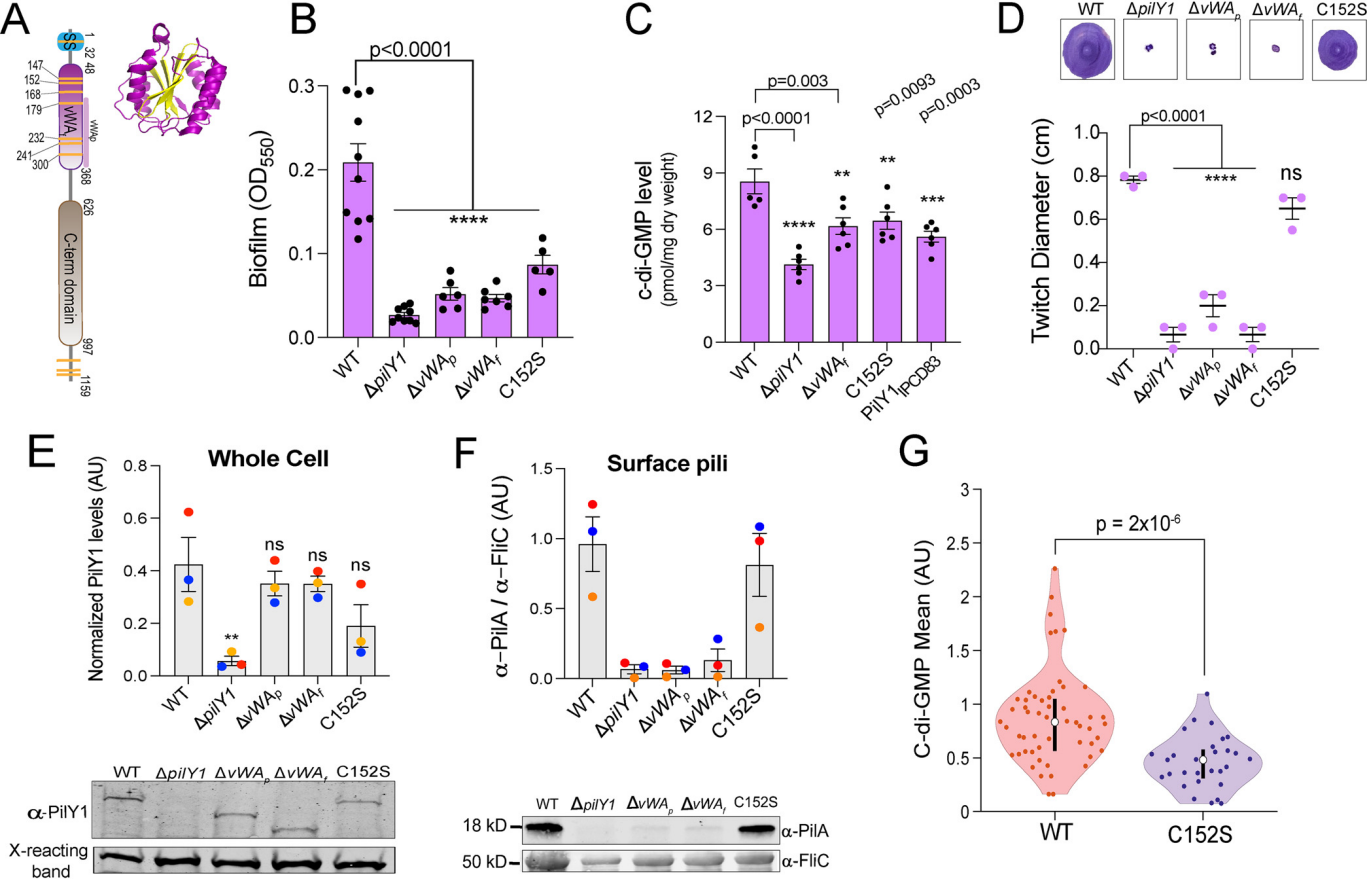
The von Willebrand A (vWA) domain and Cys152 residue of PilY1 are important for regulating c-di-GMP levels and biofilm formation. (A) Schematic showing domain organization of the PilY1 protein. The signal sequence (SS; blue, amino acids 1 to 32), vWA domain (pink, amino acids 48 to 368), and C-terminal (C-term) domain (brown, amino acids 626 to 997) are highlighted. *vWA_p_* (amino acids S178 to S365) denotes a portion of the vWA domain that is deleted from a mutant analyzed in the subsequent panels. Yellow stripes represent the cysteines residues present in the protein. The vWA domain contains 7 of the 11 cysteine residues present in the full-length PilY1 protein, with the SS and the C-terminal region having 1 and 3 cysteine residues, respectively. (Inset) Ribbon diagram showing the vWF A2 domain (PDB accession no. 3GXB). The domain shows a classical Rossmann fold (8), comprised of central β-sheets (yellow) surrounded by α-helices (purple). (B) Biofilm formation measured at an OD_550_ for the WT, the Δ*pilY1* deletion mutant, the vWA domain variants, and the Cys152S mutant in a static 96-well biofilm assay performed in M8 medium salts plus supplements (see Materials and Methods) and incubated at 37°C for 24 h. *vWA_p_* (amino acids 178 to 365 [see panel A]) and *vWA_f_* indicate a partial and a full (amino acids 48 to 368) deletion of the vWA domain, respectively. Data are from at least five biological replates, each with eight technical replicates. (C) Quantification of global c-di-GMP levels by mass spectrometry for the WT and the indicated mutants, shown in picomoles per milligram (dry weight). Cells were grown on 0.52% agar plates prepared with M8 medium salts plus supplements and then scraped from the plates after incubation for 37°C for 14 to 16 h. Data are from six biological replicates, each with two technical replicates. (D) Twitch diameter (in centimeters) for the WT and the indicated mutants measured after inoculating LB plates from overnight cultures and then incubating the plates for 24 h at 37°C plus for an additional day at room temperature. Representative images of twitch zones are shown above the graph. Data are from three biological replicates. (E) Quantification of normalized PilY1 protein levels in whole cells (in arbitrary units [AU]) for the WT and the indicated mutants. Cells were subcultured from an overnight culture and grown to mid-log phase in M8 medium salts plus supplements and normalized to the same OD_600_ value. Protein levels in whole-cell extracts are normalized to a cross-reacting band at ∼60 kDa, which is used as an additional loading control. The Cys152S mutant shows a modest but not significant reduction in PilY1 levels in whole-cell extracts. A representative Western blot image for PilY1 and the cross-reacting band is shown below the graph. (F) Quantification of normalized surface pilus levels. PilA (∼18 kDa) protein levels are used as a surrogate for surface pilus levels, which are normalized to levels of the flagellar protein, FliC (∼50 kDa). A representative Western blot is shown below the graph. All Western blot data are from three biological replicates in three independent experiments. Dots with the same color represent the same biological replicate; different colors indicate different biological replicates. *, *P* ≤ 0.05; ns, not significant. All error bars are standard errors of the means (SEM), and statistical significance was determined by one-way ANOVA and a Dunnett’s *post hoc* test. ****, *P* ≤ 0.0001; ***, *P* ≤ 0.001; **, *P* ≤ 0.01. (G) Violin plots showing the mean c-di-GMP of the WT strain and a strain expressing the vWA-Cys152S PilY1 variant during early biofilm formation. c-di-GMP level was quantified from GFP intensity, determined on a cell-by-cell basis in a microfluidic chamber for cells carrying the P*_cdrA_*-GFP construct, which is a reporter of c-di-GMP levels. Note that the WT data shown here were first reported in a previous publication (23); each strain analysis was done independently, in the same system and medium, with the same microscope at identical settings and processed as reported previously (23). Given that each analysis is independent but performed identically, we can compare data from previous studies. Each data point represents one tracked cell through an entire division cycle. Statistical significance was determined using the Kruskal-Wallis test (*P* = 2 × 10^−6^).

PilY1 is part of the priming complex together with the minor pilins that facilitate incorporation of the PilA subunits into the base of the growing pilus fiber during elongation (6, 7). During polymerization, the minor pilins and PilY1 are pushed to the tip of the growing pilus. PilY1 has a C-terminal domain that resembles PilC from *Neisseria gonorrhoea* and a N-terminal von Willebrand A (vWA) domain (Fig. 1A) that is structurally similar to the A2 domain of the human von Willebrand factor (vWF), a force-sensing glycoprotein important in stopping bleeding (4). The vWA domain of PilY1 has the classical Rossman fold—central β-sheets surrounded by amphipathic α-helices (8)—and a perfectly conserved metal ion-dependent adhesion site (MIDAS) containing the conserved DXSXS…T…D motif (9). vWA domains have been reported in TFP-associated proteins from other organisms. For example, the vWA domain of the major pilin in Streptococcus agalactiae is essential for adhesion (10), and the MIDAS motif in the vWA domain of the major pilin in Streptococcus sanguinis has recently been shown to be important in binding to eukaryotic cells (11). Like the vWF (12), the vWA domain of the P. aeruginosa PA14 PilY1 protein also has a high number of cysteine residues; 7 out of the 11 cysteines in PilY1 are in its vWA domain. Interestingly, during vascular damage, when exposed to high shear forces due to blood flow, vWF transitions from a globular to a stretched conformation (13, 14). This transition is thought to be mediated by a disulfide bond switch exposing specific sites that allow platelets to bind (15–17). Thus, vWF cysteine residues, depending on their redox state, are key to force sensing, a property that may be hypothesized for cysteine residues in the vWA domain of PilY1.

Although PilY1 is known to be important in responding to shear forces and in increasing c-di-GMP levels (3, 5, 18), how the surface signal is sensed and the precise role of its vWA domain in surface sensing and c-di-GMP signaling are unclear. Our previous genetic studies show that PilY1 and the vWA domain are important for surface-dependent stimulation of c-di-GMP production (3, 18). These studies also showed that while the C-terminal domain of PilY1 was dispensable for surface-dependent c-di-GMP production, strains with mutations in the vWA domain failed to regulate c-di-GMP levels and c-di-GMP-related behaviors (18). Additionally, deletion of the vWA domain is shown to lock PilY1 in a constitutively active signaling conformation that induces virulence independently of surface attachment (4), suggesting multiple roles for the vWA domain in the surface-attached biology of P. aeruginosa.

Recent cryo-electron tomography studies show the vWA domain of PilY1 to be situated at the very tip of the pilus fiber (6), a result consistent with the previously reported role of PilY1 as a tip-associated adhesin (19), indicating that the vWA domain is likely in immediate contact with the surface and therefore may be directly engaged in surface sensing. Based on the similarities between the human vWF and the vWA domain of PilY1 and the domain’s importance in downstream c-di-GMP signaling, we hypothesized that force-induced conformational changes originating from the vWA domain of PilY1 are mediated by conserved cysteine residues within this putative mechanosensing domain, and together these features of PilY1 are critical for surface sensing. We explore these hypotheses here.

## RESULTS

### The vWA domain of PilY1 regulates c-di-GMP levels and biofilm formation.

To address the role of the von Willebrand A (vWA) domain of PilY1 in surface sensing and c-di-GMP signaling, we made chromosomal deletions that removed a part (Δ*vWA_p_*, with amino acids S178 to S365 deleted) or all (Δ*vWA_f_*, with amino acids 48 to S365 deleted) (Fig. 1A) of the vWA domain and then performed static-biofilm assays and measured global levels of c-di-GMP (Fig. 1B and C). Our bulk assays show that both the Δ*vWA_p_* and Δ*vWA_f_* variants resulted in a significant decrease in biofilm formation and a reduction in global c-di-GMP levels (as shown for the Δ*vWA_f_* variant) compared to those of the wild type (WT). The modest but significantly increased levels of c-di-GMP for the Δ*vWA_f_* and C152S variants compared to that in the *pilY1* null strain may suggest that other parts of the N terminus of PilY1, outside the vWA domain, may be involved in surface sensing. To establish baseline biofilm levels, we included a Δ*pilA* mutant as an additional negative control (see Fig. S1A in the supplemental material).

10.1128/mbio.03754-21.1 FIG S1 (A) Biofilm formation measured at an OD_550_ for the WT, the Δ*pilY1*, Δ*pilA*, Δ*pilA* Δ*pilY1*, and vWA domain variants, and the Cys152S mutant in a static 96-well biofilm assay performed in M8 medium salts plus supplements incubated at 37°C for 24 h. The Δ*pilA* strain shows a less severe phenotype than the Δ*pilY1* strain, indicating that PilY1 has functions independent of its impact on T4P biogenesis, as previously described (3). (B and C) Partial functionality of vWA domain variants and phenotypic analysis of other cysteine vWA domain mutants. (B) Plaquing assay with phage DMS3*_vir_* versus the WT and the indicated mutants as hosts. Zones of clearing shown for the WT and the strain expressing the vWA-Cys152S mutant protein are similar, which indicates similar degrees of TFP function. The Δ*pilY1* mutant serves as the negative control. (C) Representative images of twitch zones stained with crystal violet are shown for the WT, the Δ*pilY1* mutant, or strains expressing PilY1 variants with point mutations in the Cys residues in the vWA domain following incubation at 37°C for 24 h and for one additional day at room temperature. Twitching serves as a measure of TFP function. (D) Biofilm level measured at an OD_550_ for the WT and the mutants shown in panel B using the 96-well static-biofilm assay after 24 h at 37°C, as described in Materials and Methods. Download FIG S1, PDF file, 0.2 MB. Copyright © 2022 Webster et al. 2022 Webster et al. https://creativecommons.org/licenses/by/4.0/ This content is distributed under the terms of the Creative Commons Attribution 4.0 International license.

These vWA variants also resulted in no twitching motility (Fig. 1D). To confirm these twitch phenotypes, another more sensitive assay was used based on the lysis of the P. aeruginosa PA14 host cells by the lytic DMS3*_vir_* phage, which specifically targets the TFP and also requires retraction of surface-expressed pili for infection (20). The Δ*vWA_p_* and Δ*vWA_f_* variant strains showed partial zones of clearing in a phage plaquing assay (Fig. S1B), indicating that these strains retained some TFP function. To further ensure that the decrease in biofilm formation and reduced c-di-GMP levels were not due to protein instability, we examined steady-state levels of the vWA variants in whole-cell extracts. Both the Δ*vWA_p_* and Δ*vWA_f_* PilY1 variants were stable and showed a nonsignificant reduction in whole-cell levels compared to those in the WT (Fig. 1E). However, few surface pili could be detected in the strains expressing the Δ*vWA_p_* and Δ*vWA_f_* variants (Fig. 1F), which likely explains the markedly reduced pilus function observed in the twitch assays (Fig. 1D). The presence of plaques (Fig. S1B), however, indicates that there are some surface pili, a finding consistent with our Western blot results (Fig. 1F).

### The Cys152 residue of the vWA domain is important for promoting biofilm formation and regulating c-di-GMP levels.

Multimerization and conformational changes required for function in blood clotting by the human vWF are mediated by cysteine residues (16, 17, 21). Shear forces due to blood flow during vascular damage have been shown to induce disulfide bond cleavage, which results in the protein adopting a new, stretched conformation (21, 22). Inspired by these studies and the high number of cysteines in the vWA domain of PilY1 (Fig. 1A), we hypothesized that one or more cysteines in the vWA domain of PilY1 might be important for mediating conformational changes in PilY1 and/or the pilus fiber that could in turn impact surface sensing and downstream c-di-GMP signaling. To test this hypothesis, we performed targeted mutagenesis of the cysteine residues in the vWA domain of PilY1 with the aim of identifying one or more of these residues that impact biofilm formation but still retain TFP function as assessed by twitching assays. In all cases, the mutations were introduced into the chromosomal copy of the *pilY1* gene; thus, the mutants were expressed under the native *pilY1* promoter and in their native chromosomal context. Of the seven individual and combination cysteine residues mutated, five resulted in decreased biofilm formation but no twitching motility (Fig. S1C and S1D). However, two residues, when mutated (vWA-C147S and vWA-Cys152S) displayed decreased biofilm formation but retained twitching motility (Fig. 1B and D and Fig. S1C). Because the vWA-Cys152S mutation yielded the stronger biofilm phenotype, we focused on this mutant for all subsequent analyses.

We next measured c-di-GMP levels globally and on a cell-by-cell basis for the strain expressing the vWA-Cys152S variant. Compared to the WT, the vWA-Cys152S mutant showed significantly reduced levels of c-di-GMP based on bulk measurements of cell extracts and on a single-cell basis (Fig. 1C and G, respectively).

Note that the WT data shown in the single-cell data (Fig. 1G) were first reported in a previous publication (23). Analyses of the WT and vWA-Cys152S were done independently in the same system and medium, analyzed with the same microscope at identical settings, and processed as reported previously (23). Given that each investigation was independent but performed identically, it allowed us to compare data from this previous report.

Given the similar levels of twitching motility for the strain carrying the vWA-Cys152S mutant and the WT, we predicted that this point mutation would yield a stable PilY1 protein. Western blot studies of whole cells showed that the vWA-Cys152S variant is stable and shows a modest but nonsignificant reduction in protein levels compared to those of WT PilY1 (Fig. 1E). Additionally, surface pilus levels for the strain expressing the vWA-Cys152S mutant protein are comparable to those of the WT (Fig. 1F). These results are consistent with the vWA-Cys152S mutant showing levels of twitching motility (Fig. 1D) and plaque formation (Fig. S1B) similar to those of the WT. Of note, none of the observed phenotypes are due to differences in growth rates, as the vWA-Cys152S strain, along with all vWA domain mutants used in this study, has the same growth kinetics as the WT (Fig. S2).

10.1128/mbio.03754-21.2 FIG S2 Growth curves for the WT and the strains expressing the PilY1 variants. Growth assays were performed in M8 minimal salts medium supplemented with Casamino Acids, glucose, and magnesium sulfate. This medium was also used for all macroscopic biofilm assays, c-di-GMP measurements, plaquing assays, and AFM studies. The data are from three biological replicates, each with two technical replicates. There is no significant difference among the growth kinetics of all strains. Error bars show SEM, and statistical significance was determined at each time point using one-way analysis of variance (ANOVA) and the multiple-comparison test. Download FIG S2, PDF file, 0.1 MB. Copyright © 2022 Webster et al. 2022 Webster et al. https://creativecommons.org/licenses/by/4.0/ This content is distributed under the terms of the Creative Commons Attribution 4.0 International license.

### The vWA-Cys152S variant of PilY1 is associated with lower surface adhesion forces and altered force-induced behaviors.

**FIG 2:**
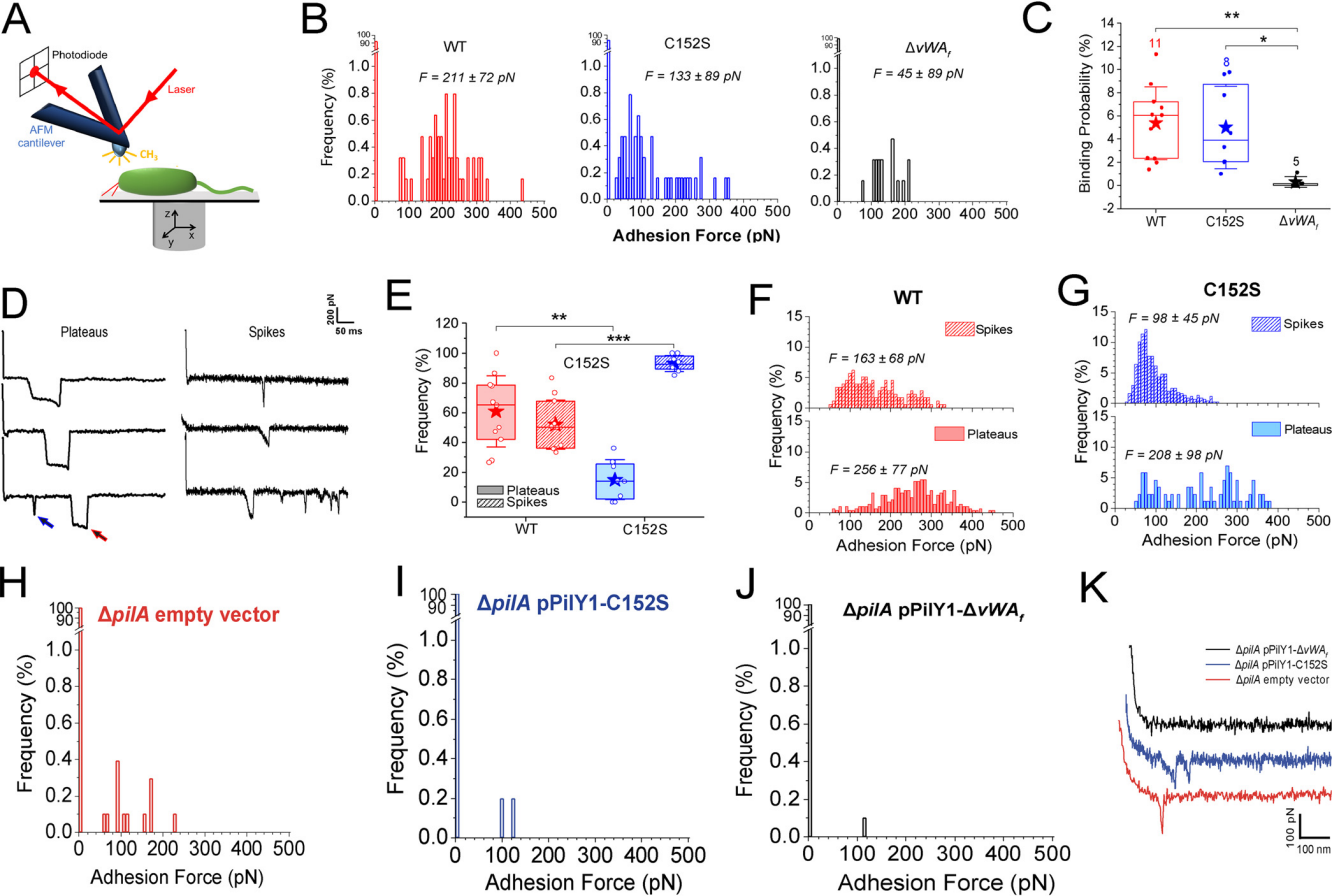
Strains expressing the PilY1-Cys152S mutation display less adhesion force and altered mechanical behaviors than strains expressing WT PilY1. (A) For AFM measurements, it is required that probed cells are immobile. To immobilize the cells, we used a hydrophobic polystyrene surface that allowed for sufficient binding of the bacterium to the surface through its cell envelope to immobilize the cell. Force-distance curves were recorded in a square array at a bacterial pole (see Materials and Methods), because the pili are localized to the pole, which involved sequentially approaching the AFM tip toward the cell pole, making contact, and then retracting the tip. Adhesive interactions occurring between the pilus and the AFM tip upon contact allow catching and subsequently stretching the pilus as the AFM tip is retracted, which causes a deflection of the cantilever. This deflection is recorded by a laser beam and photodiode and is directly proportional to tensile force. The tensile force in the stretched pili can be determined from the generated force-distance curves. (B) Adhesion force histograms between the hydrophobic AFM tip and a representative WT strain or strains expressing the Cys152S or Δ*vWA_f_* variants of PilY1. For the WT, 211 ± 72 pN (*n* = 55 adhesive curves); for the vWA-Cys152S, 133 ± 89 pN (*n* = 47); and for the Δ*vWA_f_*, 45 ± 89 pN (*n* = 16). (C) Box plots comparing the binding probabilities of cells expressing WT PilY1 and of strains expressing the Cys152S or Δ*vWA_f_* variant of PilY1 are shown. The numbers of probed cells are indicated. Stars are the mean values, lines the medians, boxes the 25 to 75% quartiles, and whiskers the standard deviations (SD). *, *P* ≤ 0.05; **, *P* ≤ 0.01 (Student's *t* test). (D) Representative retraction force profiles exhibited by the WT or Cys152S mutant cells sorted based on their shape. Plateaus are defined as adhesive events with a “step” behavior, i.e., a constant sustained force over a defined length of time, while spikes are defined as sharp adhesive events with a single minimum. A single retraction profile can feature several plateaus (red arrow) and spikes (blue arrow), and both signatures can occur as marked by the arrows. (E) Box plots comparing the occurrences of plateau (shaded) and spike (striped) signatures for the WT and Cys152S mutant cells. The number of probed cells is as described for panel C. For the WT, plateaus were 60.8% ± 24.0% and spikes were 51.9% ± 16.6% (*n = *11), and for Cys152S mutant, plateaus were 14.9% ± 13.3% and spikes were 93.1% ± 5.4% (*n = *8). Stars are the mean values, lines the medians, boxes the 25 to 75% quartiles, and whiskers the SD. **, *P* ≤ 0.01; ***, *P* ≤ 0.001 (Student's *t* test). (F and G) Distribution of the adhesion forces exhibited by either the plateaus or the spikes for the WT (F) or the strain carrying the Cys152S mutant of PilY1 (G). The mean values are provided along with the histograms. All data were obtained by recording force-distance curves in medium containing M8 salts with an applied force of 250 pN and a pulling speed of 5 μm/s at room temperature. (H to K) The pilus fiber is required for adhesion to a surface. (H to J) Adhesion force histograms obtained by recording force-distance curves between the hydrophobic cantilever tip and representative Δ*pilA*/pPilY1 (H), Δ*pilA*/pPilY1-Cys152S (I), and Δ*pilA*/pPilY1-ΔvWA_f_ (J) strains. (K) Representative retraction force profiles shown for the same strains.

In light of the key role of the vWA domain in biofilm formation and c-di-GMP regulation, we next sought to investigate the different surface adhesion behaviors of P. aeruginosa strains expressing WT PilY1 or the PilY1 variants with the Δ*vWA_f_* or the vWA-Cys152S mutation. To this end, we used atomic force microscopy (AFM), a powerful multifunctional technique that has been instrumental in deciphering the adhesion and nanomechanical properties of bacterial pili, at the single-cell and single-molecule levels (24–26). More specifically, we recorded the force experienced by a hydrophobic AFM tip when probed against the TFP of surface-engaged bacterial cells as a function of the tip-to-sample surface distance (note that bacterial cells are immobilized on a surface, and the pili are free to contact the tip of the cantilever) (Fig. 2A). From the resulting force-distance curves, binding probability and adhesion forces were determined for multiple living cells. As illustrated in the representative force histograms (Fig. 2B), the vWA-Cys152S mutant showed a lower adhesion force (*F*) than WT cells (*F* = 133 ± 89 pN and 211 ± 72 pN, respectively; *P* < 0.001), indicating that the Cys152S mutation reduces the interaction strength. However, both WT and vWA-Cys152S PilY1 cells showed similar probabilities of binding to the hydrophobic AFM tip (Fig. 2C), a result that is consistent with both strains having similar levels of surface pili (Fig. 1G). Cells with the full deletion of the vWA domain (Δ*vWA_f_*) showed a probability of binding to the hydrophobic tip close to zero (Fig. 2C) and a low adhesion force (∼45 pN) (Fig. 2B), likely due to the small number of surface pili (Fig. 1F); alternatively, a similar finding may be observed if the pili are shorter in length. These data suggest that the observed force curves are dependent on TFP-associated PilY1.

For cells expressing WT PilY1 and the vWA-Cys152S variant, both of which showed adhesion to the hydrophobic AFM tip, two distinct adhesive behaviors were observed: plateaus (red arrow in Fig. 2D) and spikes (blue arrow in Fig. 2D). Plateaus are defined as adhesive events with a “step” behavior, that is, a constant sustained force over a defined length of time, while spikes are defined as sharp adhesive events with a single minimum and are reflective of a nanospring behavior (26). Plateaus relate to sequential increases in length (structural changes) that occur over discrete segments of the pilus fiber (including the tip protein PilY1) at a constant tension, as described previously (27), while spikes arise from more uniform (or cooperative) stretching across the length of the pilus, including PilY1. Thus, the mutation in Cys152 of the PilY1 tip protein alters the overall behavior of the pilus in terms of localized versus distributed conformational changes. Furthermore, plateaus and spikes are not mutually exclusive in their appearance and frequency. Cells expressing WT PilY1 or the vWA-Cys152S variant showed plateaus and spikes; however, the frequencies of these behaviors differed significantly between the strains (Fig. 2E). Cells expressing WT PilY1 had similar proportions of plateaus (∼61%) and spikes (∼52%; the sum can be >100% because some force curves can have both features). In contrast, cells expressing the vWA-Cys152S mutant of PilY1 showed a significantly lower frequency of plateaus (∼15% compared to ∼61% for the WT) and a much higher frequency of spikes (∼93% compared to ∼52% for the WT) (Fig. 2E). These data indicate that a single point mutation in the PilY1 vWA domain can have a marked impact on the cell’s mechanical behavior.

Finally, the magnitudes of the adhesive signatures for both spikes and plateaus were higher for cells with WT PilY1 than those for cells expressing the vWA-Cys152S variant (Fig. 2F and G), consistent with the observation that cells expressing WT PilY1 can sustain globally higher adhesive forces than the cells expressing the vWA-Cys152S mutant (Fig. 2B). Interestingly, for both strains, the observed plateau forces are higher than those observed for the spikes, which, along with the higher frequency of plateaus observed in WT PilY1, also explains the higher forces sustained by the WT cells.

Our data above indicate that the observed adhesive forces as well as the plateau and spike signatures observed for strains expressing the WT PilY1 protein versus the vWA-Cys152S mutant protein were dependent on PilY1 and its vWA domain. We next asked where these force profiles were dependent on the TFP. Because PilY1 is cell surface associated and can be secreted to the cell surface independently of the TFP machinery (3), we expressed plasmid-borne WT PilY1 and the vWA-Cys152S mutant protein in a Δ*pilA* background, lacking the full pilus fiber, and performed AFM experiments. Both the strain expressing the WT PilY1 protein and the strain expressing the vWA-Cys152S mutant protein showed little adhesion to the hydrophobic tip (binding probability < 1%) (Fig. 2H to J). The adhesive events recorded for the strains expressing these proteins in the Δ*pilA* background were significantly lower than those exhibited when the pilus was present, and plateau signatures were never observed (Fig. 2K). Instead, typical receptor-ligand signatures were recorded, resembling a spike signature but with a very short rupture length, consistent with the length of the protein that is stretched while the AFM tip retracts away from the bacterium (Fig. 2H to J). Together, the genetic and AFM data support the hypothesis that the adhesive forces measured, as well as the plateau and spike signatures exhibited by the strains expressing the WT PilY1 protein and the vWA-Cys152S mutant protein, are due to both PilY1 and the pilus fiber.

### The vWA-Cys152S mutation has a negligible impact on the solution conformation of the vWA domain.

**FIG 3:**
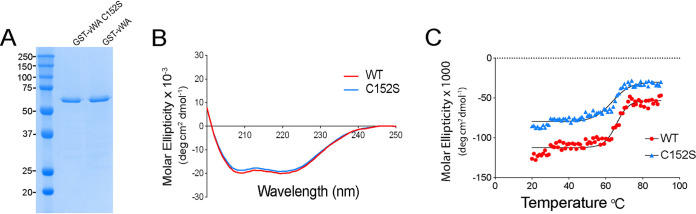
vWA-C152S mutation does not substantially alter conformation of the vWA domain in solution. (A) Coomassie blue-stained SDS-PAGE of ∼1 μg of purified wild-type GST-vWA domain and GST–vWA-C152S fusion proteins expressed from a pGEX plasmid backbone and purified from E. coli BL21(DE3) cells as detailed in Materials and Methods. The molecular weight markers are indicated. (B) Far-UV circular dichroism (CD) spectra shown in molar ellipticity for the WT GST-vWA domain (red line) and GST–vWA-C152S mutant (blue line) between 195 and 250 nm at 20°C. (C) Curves of ellipticity at a 208-nm wavelength as a function of temperature for the WT and mutant fusion proteins. Spectra were recorded for each sample from 20 to 90°C in 1° increments. Curves were fitted to a Boltzmann sigmoidal equation, and the *V*_50_ value (mid-point of the slope) was determined (65.8 versus 63.5°C for the GST-WT vWA domain and GST–vWA-C152S fusion variant, respectively).

Given our findings of the significant difference in mechanical behaviors observed for the strains expressing the WT PilY1 protein and the vWA-Cys152S mutant protein when these strains are engaged with a surface and thus under mechanical tension, we next determined whether this single cysteine mutation affected the conformation of the purified, isolated vWA domain of PilY1 in solution. We focused on the vWA domain because, despite attempts with several different expression systems, we were unable to purify stable, full-length PilY1 or the N-terminal domain of this protein. We cloned the WT vWA domain and vWA-C152S (amino acids 30 to 369) as glutathione *S*-transferase (GST) fusion proteins to enhance stability and facilitate purification. A GST domain and an HRV-3C protease cleavage site were added to the N terminus of the vWA domain protein, and the resulting fusion proteins were overexpressed in Escherichia coli cells and purified to apparent homogeneity (Fig. 3A). The HRV-3C protease cleavage site was confirmed by sequencing. Unfortunately, repeated attempts to efficiently cleave the GST domain from the vWA domain proteins with protease HRV-3C were unsuccessful, perhaps due to steric occlusion of the protease binding site in the purified proteins. Thus, the studies described below were done using GST-vWA domain fusion proteins.

We performed far-UV circular dichroism (CD) spectroscopy to determine the secondary structures of the WT and mutant fusion proteins and to assess the thermal stability of the WT vWA domain protein and the vWA-Cys152S variants (Fig. 3B and C). Far-UV CD spectra of the GST-WT vWA domain and GST–vWA-Cys152S fusion proteins were monitored at wavelength scans between 195 and 250 nm. Both the WT and mutant spectra showed the presence of two distinct negative peaks centered at 208 and 222 nm, typical of α-helical proteins (Fig. 3B). Overall, the dichroic spectra for the GST-WT vWA domain protein and GST–vWA-Cys152S were similar. Measuring CD as a function of temperature can be used to determine the effects of mutations on protein stability. Analysis of the ellipticity curves in the range of 20 to 90°C showed the melting temperatures of the GST-WT vWA domain protein and the GST–vWA-C152S fusion variant to be similar (65.8 versus 63.5°C) (Fig. 3C), suggesting that the C152S mutation did not perturb the secondary structure of the domain in solution (i.e., in the absence of mechanical force).

### Genomic analyses reveal that PAO1 strains lack the vWA-C147 and vWA-Cys152 cysteine residues that are present in PA14 strains, with associated functional consequences.

**FIG 4:**
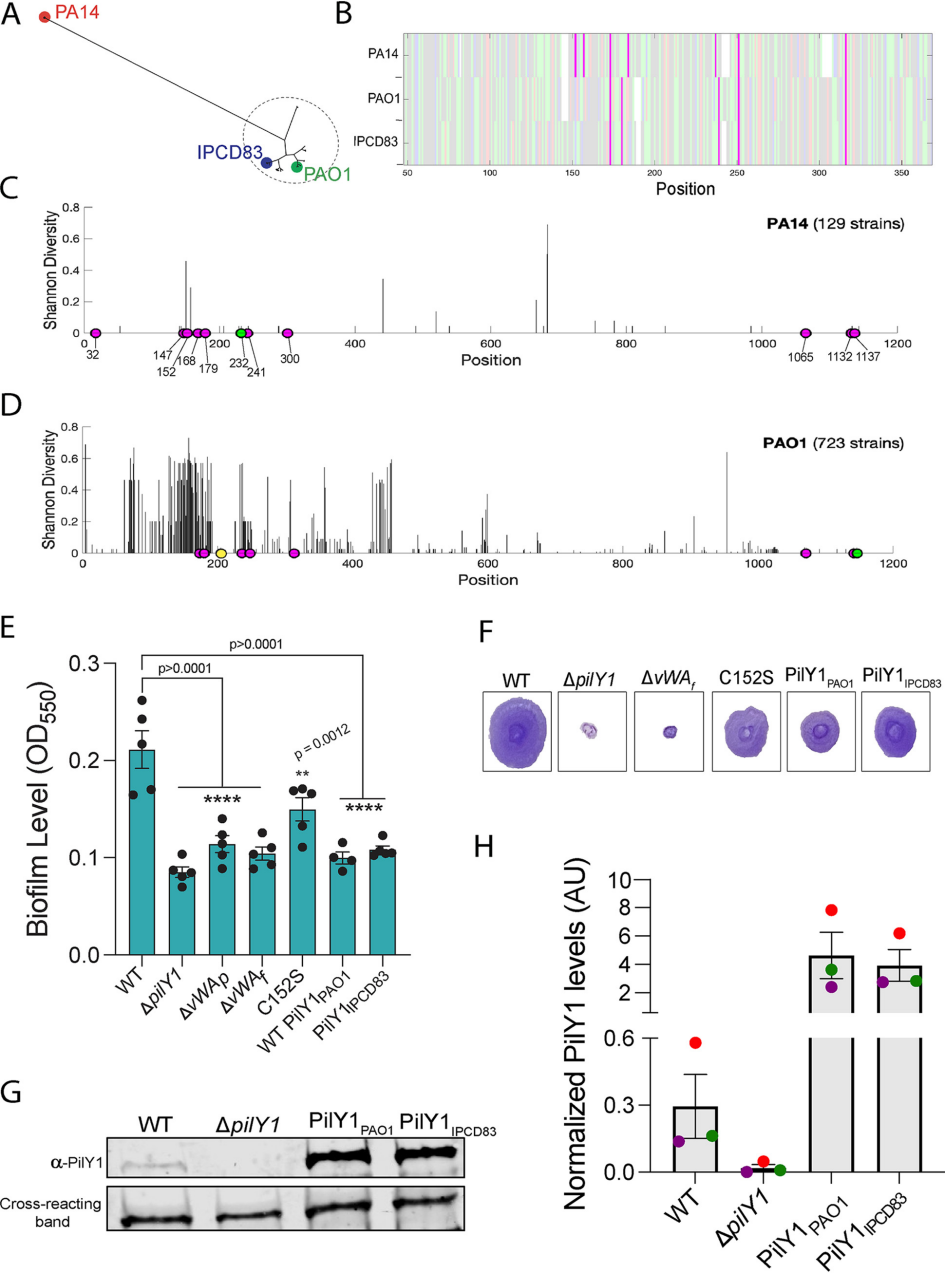
Comparative genomic analyses reveal sequence and functional differences between PA14 and PAO1 alleles of PilY1. (A) Phylogenetic tree of PilY1 amino acid sequences obtained from the IPCD database of P. aeruginosa genomes (28) showing two distinct clades of PilY1 sequences corresponding to strains from the previously determined P. aeruginosa PA14 and PAO1 clades. The strain labeled IPCD83 is an isolate within the PAO1 clade. (B) Alignment of the vWA domain (amino acids 48 to 368) of PilY1 proteins found in the PA14, PAO1, and IPCD83 strains, with cysteines highlighted in magenta. Positive, negative, polar, and hydrophobic amino acids are depicted in light pink, blue, green, and gray, respectively. (C) Shannon diversity index along the PilY1 amino acid sequence for the 129 PilY1 proteins belonging to the PA14 clade. Fully conserved cysteines are highlighted in magenta. One strain is missing the cysteine depicted in green. (D) Shannon diversity index along the PilY1 amino acid sequence for the 723 versions of PilY1 proteins belonging to the PAO1 clade. Fully conserved cysteines are highlighted in magenta. One strain is missing the cysteine depicted in green, and one strain has an extra cysteine depicted in yellow. (E) Biofilm formation measured at an OD_550_ in a static 96-well assay for the indicated strains. Hybrid P. aeruginosa PA14 strains carry the PilY1 protein from PAO1 (PilY1_PAO1_) or the PilY1 protein from the IPCD83 strain (PilY1_IPCD83_), replacing the coding region for the P. aeruginosa PA14 PilY1 protein. In all cases, the mutant PilY1 variants are expressed from the native locus of P. aeruginosa PA14. Error bars are SEM, and statistical significance shown was determined by one-way ANOVA and Dunnett’s *post hoc* test. ****, *P* ≤ 0.0001; ***, *P* ≤ 0.001; **, *P* ≤ 0.01; ns, not significant. (F) Representative images of twitch zones shown for the indicated strains. (G) Representative Western blot image for steady-state PilY1 protein levels in whole cells expressing WT PilY1, the Δ*pilY1* mutant, PilY1_PAO1_, and PilY1_IPCD83_. (H) Quantification of normalized PilY1 protein levels from whole cells for strains shown in panel G. Protein level is normalized to a cross-reacting band at ∼60 kDa. Data are from three biological replicates in three independent experiments. Dots with the same color represent the same biological replicate; different colors indicate different biological replicates.

Given our findings that cells expressing the vWA-Cys152S mutation impact surface sensing, c-di-GMP levels, and biofilm formation (Fig. 1B and C; Fig. S1C) in P. aeruginosa PA14, we analyzed whether the Cys152 residue was conserved across P. aeruginosa strains. We leveraged PilY1 sequences from the international P. aeruginosa consortium database (IPCD), a repository for thousands of P. aeruginosa isolates from a diverse range of environments (28). We analyzed the phylogenetic relationship of PilY1 amino acid sequences from a total of 852 P. aeruginosa genomes and found two distinct clades, PA14 (red dot in Fig. 4A) and PAO1 (green dot, dashed circle in Fig. 4A), largely consistent with a previous report by Freschi and colleagues (28). The PilY1 sequence from the strain IPCD83 (blue dot) falls within the PAO1 clade. Alignment of the amino acid sequences of the vWA domain of PilY1 from the PA14, PAO1, and IPCD83 strains shows that five of the seven cysteines (magenta) in the vWA domain of PA14 are highly conserved in PAO1 and IPCD83, although the spacing of the residues varies in some cases (Fig. 4B). All three domains consist of positive, negative, polar, and hydrophobic amino acids shown in light pink, blue, green, and gray, respectively. Of note is the high abundance of polar residues in the vWA domains of all three strains.

To examine the amino acid diversity of the PilY1 sequences in the PA14 and PAO1 clades, we computed Shannon diversity indices as a measure of sequence diversity (Fig. 4C and D). We aligned PilY1 sequences within the PA14 (Fig. 4C) and PAO1 (Fig. 4D) clades and calculated the Shannon diversity at each amino acid position. As shown in Fig. 4C, there is very little amino acid sequence diversity over the entire PilY1 sequence among the 129 isolates with PA14 versions of PilY1. Interestingly, all strains in the PA14 clade except one contain the 11 cysteines (magenta circles) found in the PA14 reference strain (Fig. 4C). Furthermore, each isolate had all seven cysteines in the vWA domain, while there was one strain missing the vWA-C232 residue (a residue that we found to be critical for TFP-mediated twitching motility) (green circle in Fig. S1C). In contrast to strains in the PA14 clade, strains within the PAO1 clade showed low diversity at the C-terminal domain (amino acids 626 to 997) and high amino acid diversity in the vWA domain (amino acids 48 to 368) (Fig. 4D). Additionally, of the 723 variants of the PilY1 sequences from the PAO1 clade analyzed, only 8 cysteines were highly conserved, compared to the 11 highly conserved cysteines for the PA14 strains. The vWA domain of the PAO1 clade contains five of the seven conserved cysteines found in the PA14 clade. Interestingly, vWA-C147 and vWA-Cys152 residues are not present in any of the PAO1 strains, including the IPCD83 isolate. Recall that we showed that both vWA-C147 and vWA-Cys152 residues are important in c-di-GMP signaling, and mutations in these residues resulted in strains that had decreased biofilm formation but that retained twitching motility (Fig. 1B and Fig. S1C).

Given the biofilm phenotype of the strain expressing the vWA-Cys152 variant of PilY1 (Fig. 1B and Fig. S1C) and the role of PilY1 in early biofilm formation and c-di-GMP signaling, we expected that the loss of the vWA-Cys152 residue in strains from the PAO1 clade, including IPCD83, would result in similar phenotypes. To test this hypothesis, we cloned the *pilY1* gene from the IPCD83 isolate (PilY1_IPCD83_) or the WT PAO1 strain (WT PilY1_PAO1_) into the native locus of the reference PA14 strain and performed static-biofilm assays. As with the vWA-Cys152S mutation, both PAO1 variations expressed in the PA14 strain resulted in significantly decreased levels of biofilm formation compared to that of the WT (Fig. 4E). Quantification of c-di-GMP levels for PilY1_IPCD83_ showed a significant decrease in c-di-GMP level (Fig. 1C). Additionally, both the PilY1_PAO1_ and PilY1_IPCD83_ variants still supported twitching motility at a level that is similar to the level for the vWA-Cys152S mutant protein (Fig. 4F). The PilY1_PAO1_ and PilY1_IPCD83_ variants showed levels of PilY1 expression that exceeded levels in the WT (Fig. 4G and H), indicating that the observed phenotypes were not due low-level expression of these variants.

## DISCUSSION

Our data show that force-induced changes mediated by one or more cysteine residues in the vWA domain of the TFP tip-associated protein PilY1 are required for surface sensing and downstream c-di-GMP signaling and biofilm formation. The concepts of mechanical force inducing protein conformational changes, that these changes are modulated by disulfide bonds, and that such changes in conformation are required for function are well studied for the eukaryotic proteins titin and vWF. Titin undergoes cycles of folding and refolding that allow it to function as a molecular spring during cycles of muscle relaxation and contraction, respectively (29, 30). When force is applied, the immunoglobin (Ig) domains of titin unfold and extend (31). Similarly, increased shear forces due to blood flow cause the vWF protein to transition from a globular to a stretched conformation (32); this stretched conformation allows the vWF protein to bind to platelets and form a clot at sites of vascular damage (33). Furthermore, the folding and refolding events observed for titin and the vWF protein are mediated by disulfide bonds (34, 35). For titin, oxidation of the disulfide bond greatly increases both its speed and its magnitude of folding (36), while the redox state of the disulfide bond in the A2 domain of the vWF protein determines the exposure of platelet binding sites (21). Additionally, disulfide bonds in FimH, the adhesin on the type I pilus in E. coli (37), are essential for adhesion in high-flow environments (38).

The vWA domain of PilY1 in P. aeruginosa PA14 has seven cysteine residues. Our genetic analyses show that two of these residues, vWA-Cys152 and to a lesser extent vWA-Cys147, are critical for PilY1-dependent surface signaling and biofilm formation. The vWA-Cys152 mutation does not destabilize the PilY1 protein. Our AFM studies support the conclusion that strains expressing the vWA-Cys152S mutant result in cells that are still capable of surface attachment at the same frequency as that of the WT. Using AFM, we show that the WT cells display spike signatures which are typical of nanospring behaviors (26). That is, TFP/PilY1 can display elastic properties upon the application of force, but once the force is removed, the pilus rapidly returns to its original conformation. Based on previous work (26) and our data here, these force profiles appear to require both TFP and PilY1. Such nanospring properties are also observed for SpaC, a vWA domain-containing protein that is a key pilus-associated adhesin of Lactobacillus rhamnosus GG (24). Under high mechanical forces, SpaC is shown to behave like a spring. This spring-like behavior is thought to allow the bacterium to withstand higher forces under shear stress when the pilus is stretched and presumably allows the pilus to engage the surface under strain without snapping (24).

The WT P. aeruginosa PA14 strain also shows plateau signatures. One interpretation of these plateau signatures is that they reflect the pilus being bound to the surface at multiple points, followed by successive desorption of the pilus (39). Alternatively, plateau signatures may be indicative of sustained protein conformational changes. In either case, the plateaus observed for WT cells produce high adhesive force signatures and thus likely help to promote surface engagement.

We found that mutating the Cys152 residue of the vWA domain of PilY results in a reduction in biofilm formation and lower levels of c-di-GMP production. A strain expressing this mutant variant also shows significant changes in mechanical properties (detailed below) when the cell is subjected to force. That these changes in mechanical behavior are dependent on applying a force is in line with our CD and melting curve data, which show no differences in the overall global and secondary structures for the WT and the Cys152Ser variant when in solution (i.e., in the absence of an applied force).

The findings from our AFM analysis of the WT and vWA-Cys152Ser variant of the PilY1 protein raise some interesting implications. The ∼50/50% distribution of plateaus and spikes observed in cells with WT PilY1 may suggest a built-in property that allows for inherent heterogeneity in surface adaptation. That is, transient changes in PilY1 conformation (the spike signatures) may not be sufficient to drive signaling; only sustained conformational changes (i.e., plateaus) can do so. Our observations that the vWA-Cys152S mutant variant of PilY1 is skewed ∼90:10 toward spike signatures (i.e., transient conformational changes) and that this strain is defective for c-di-GMP signaling support this conclusion. Thus, not every interaction between a cell and the surface to which it might attach is “productive,” a conclusion consistent with several reports showing the heterogenous nature of P. aeruginosa populations transitioning to a biofilm lifestyle (23, 40–42). Furthermore, we could predict then that a PilY1 mutant that favors the plateau conformation promotes c-di-GMP signaling and is a hyper-biofilm former. We have performed extensive genetic screens looking for PilY1 mutants with such phenotypes with no success to date. Thus, an alternative explanation is that the ability of TFP/PilY1 to transition between conformations is key to the ability to signal properly and that locking the protein in one conformation, or another, results in aberrant signaling.

The critical role for vWA-Cys152 in c-di-GMP signaling and biofilm formation is supported by our genomic analysis, which highlights differences in the PilY1 protein among PA14 and PAO1 strains. The vWA domains of PilY1 from the PA14 and PAO1 strains are very different, with PilY1 proteins from the PAO1 clade (PAO1 and IPCD83) lacking the conserved vWA-Cys152 and vWA-Cys147 residues. P. aeruginosa PA14 strains engineered to carry the PAO1 or IPCD83 alleles of the gene for PilY1 which lacks the conserved vWA-Cys152 and vWA-Cys147 residues result in a hybrid strain that behaves very much like the P. aeruginosa PA14 strain carrying the vWA-Cys152S mutant protein. Thus, our genetic analysis confirmed that the observed sequence differences have functional consequences. The distinct PilY1 proteins of P. aeruginosa PA14 and PAO1 may also contribute to explaining the differences in the surface commitment strategies observed for these strains, as reported previously (3, 41).

Our AFM data show that force curve plateaus can be maintained for up to 50 ms; it is important to note that this value may be an underestimation because desorption of the pilus from the AFM tip may result in the force curve returning to baseline. With this important caveat in mind and considering that the P. aeruginosa TFP has a known retraction rate of ∼0.5 μm s^−1^ (43), then the distance that the TFP is retracted during this 50-ms window (the time that span plateaus are maintained) is ∼0.025 μm. This is quite a short distance (TFP can exceed 2 μm) and corresponds to the pilus being (almost) fully retracted, with the priming complex (i.e., the minor pilins) and the vWA domain of PilY1 docked into the pore of the secretin (6). Furthermore, if we postulate that TFP/PilY1-mediated signaling is a consequence of mechanical force, for the TFP/PilY1 to remain under force and thus potentially capable of propagating a signaling event via a conformational change, we hypothesize that at least one other pilus needs to be bound to the surface to pull in opposition to the fully retracted pilus described above. That is, PilY1-mediated signaling would require multiple pili to decorate the cell surface; this model has a key corollary in that TFP must be robustly expressed for signaling to occur. Interestingly, previous studies (44–47) and work from our team (3) show that the levels of TFP are low in planktonic cells. Furthermore, our team showed that increased cAMP levels via the Pil-Chp system (3), which is key for pilus production, might require several cellular generations and multiple transient surface interactions to occur (41). Thus, our previous observations of a role of multigeneration cAMP signaling via TFP may be necessary to produce multiple TFP; multiple TFP, in turn, are required for subsequent c-di-GMP signaling.

**FIG 5:**
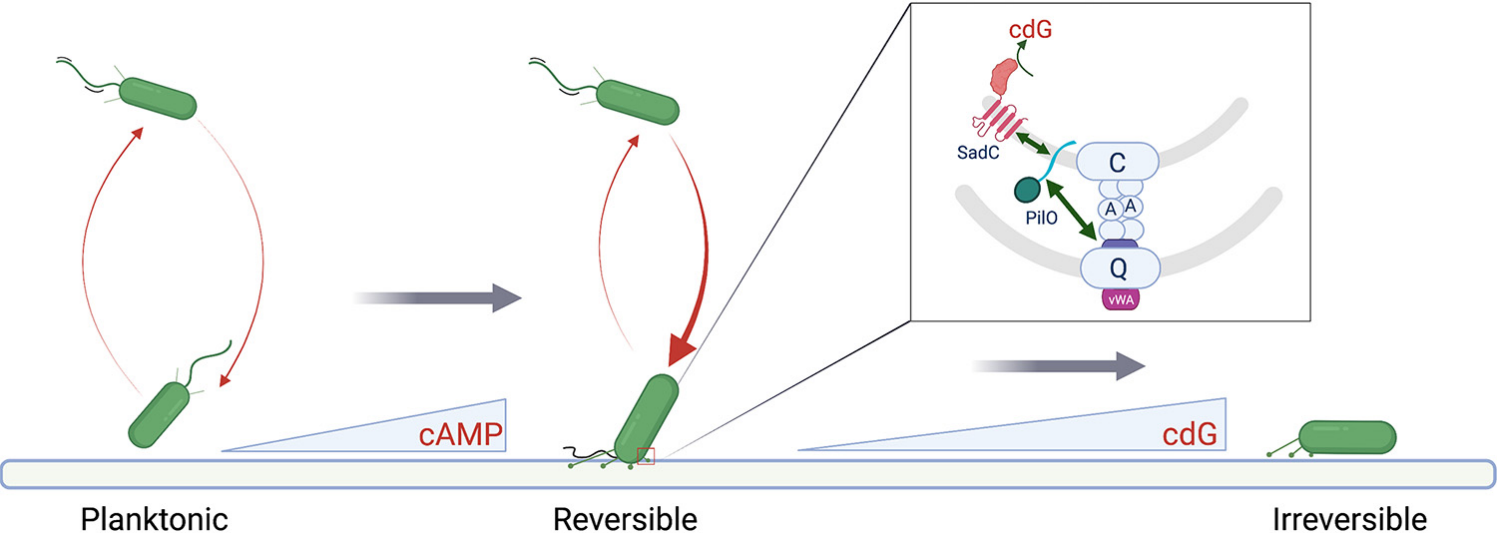
Proposed model for mechanical force driven transition from planktonic to irreversible attachment. Planktonic bacteria interact with the surface and increase cAMP levels and surface pilus levels. The proposed PilY1-PilO interaction can in turn drive the documented PilO-SadC signal transduction cascade, which stimulates c-di-GMP (cdG) signaling and increased biofilm formation.

Based on the data presented here and previous studies from our team and others (3, 23, 41), we propose the following model of the early events initiating biofilm formation by P. aeruginosa PA14 (Fig. 5). When the TFP of P. aeruginosa PA14 initially engage the surface, we propose that the Pil-Chp signaling cascade promotes cAMP production, which in turn enhances transcription and subsequent production of TFP over the low levels of these appendages produced by planktonically grown bacteria (3). Currently, we do not have a strong mechanistic understanding of the linkage(s) among TFP, the Pil-Chp system, and cAMP production. However, once more pili are deployed to the surface, this event provides the necessary condition for multiple surface-engaged TFP working in opposition to generate mechanical tension. This mechanical tension in turn can drive the sustained, PilY1-Cys152-dependent conformational changes that we have observed for WT cells. That is, the conformational change in the vWA domain of PilY1 is maintained only during the application of force when the TFP pull against a solid surface and thereby generate tension (with the cells presumably not moving). We propose that when multiple TFP engage the surface, the change in TFP/PilY1 conformation can be sustained as the pilus retracts and PilY1 is docked in the PilQ pore; here, PilY1 can interact with PilO, as has been reported for the homologous system in Myxococcus xanthus (6). Based on our recent study (23), the proposed PilY1-PilO interaction can in turn drive the documented PilO-SadC signal transduction cascade (23), which stimulates c-di-GMP signaling and increased biofilm formation. It is also important to note that a recent pulldown analysis indicates that there is one molecule of PilY1 per pilus in M. xanthus (6); thus, it is unlikely that intermolecular disulfides are being formed with other PilY1 proteins. Additionally, cryo-electron tomography shows the C-terminal domain of PilY1 to be in direct contact with the minor pilins while the vWA domain is at the apex of the pilus fiber (6), suggesting that intermolecular disulfide bond formation between PilY1 and any of the minor pilins is also unlikely. Consistent with this conclusion, our purification of the vWA domain and Western analysis of cell surface PilY1 show no evidence of PilY1 forming intermolecular multimers.

Finally, our studies were able to successfully separate the role of TFP in motility from their role in signaling. Work from our team and others (2, 3, 18, 23, 48) has implicated TFP in surface sensing via the surface-dependent stimulation of the second messengers cAMP and c-di-GMP; however, in these studies, the mutants used also disrupted TFP-mediated twitching motility. Here, the Cys152S allele of PilY1 results in decreased c-di-GMP signaling, but strains carrying this mutation display robust twitching motility. In consideration of our previous studies showing a role of the TFP alignment complex component PilO in c-di-GMP production (23), we believe that it is quite clear that TFP are not only key appendages for adhesion and surface motility but also central players in surface-specific signal transduction.

## MATERIALS AND METHODS

### Bacterial strains, plasmids, media, and growth conditions.

All bacterial strains used in this study are listed in Table S1 in the supplemental material. P. aeruginosa PA14 and E. coli S17-λ-pir were routinely grown in 5 ml lysogeny broth (LB) medium or struck on 1.5% LB agar plates with appropriate antibiotics, if necessary. Overnight cultures were grown in LB at 220 rpm on a roller drum. Saccharomyces cerevisiae InvSc1 (Thermo Fisher) used for cloning was maintained on yeast peptone dextrose (YPD; 1% Bacto yeast extract, 2% Bacto peptone, and 2% dextrose) with 2% agar. Synthetic defined medium without uracil (Sunrise Science Products) was used to select for yeast with the construct. All chromosomal point mutations were constructed using the pMQ30 shuttle vector, while the pMQ72 multicopy plasmid was used for ectopic expression. All plasmids and oligonucleotides used in this study are listed in Tables S2 and S3, respectively.

10.1128/mbio.03754-21.3 TABLE S1 Strains used in this study. Download Table S1, PDF file, 0.08 MB. Copyright © 2022 Webster et al. 2022 Webster et al. https://creativecommons.org/licenses/by/4.0/ This content is distributed under the terms of the Creative Commons Attribution 4.0 International license.

10.1128/mbio.03754-21.4 TABLE S2 Plasmids used in this study. Download Table S2, PDF file, 0.1 MB. Copyright © 2022 Webster et al. 2022 Webster et al. https://creativecommons.org/licenses/by/4.0/ This content is distributed under the terms of the Creative Commons Attribution 4.0 International license.

10.1128/mbio.03754-21.5 TABLE S3 Oligonucleotides used in this study. Download Table S3, PDF file, 0.06 MB. Copyright © 2022 Webster et al. 2022 Webster et al. https://creativecommons.org/licenses/by/4.0/ This content is distributed under the terms of the Creative Commons Attribution 4.0 International license.

### Construction of deletion mutant strains.

All in-frame chromosomal gene deletions were constructed using the pMQ30 shuttle vector carrying the flanking regions of the gene by homologous recombination using the yeast machinery (49) or by Gibson cloning as previously described in reference 50. For yeast cloning, S. cerevisiae was grown overnight at 30°C in YPD synthetic defined medium without uracil (Sunrise Science Products), which was used to select for yeast colonies with the plasmid construct. Plasmids were extracted from yeast using the “smash and grab” method, transformed by electroporation into E. coli S17 cells, and grown on LB plates with 10 μg/ml gentamicin at 30°C overnight (2). Colony PCR amplification and sequencing were used to confirm plasmid construction. Plasmids were introduced in P. aeruginosa by conjugation, and merodiploids were selected on 25 μg/ml gentamicin and 20 μg/ml nalidixic acid, after which cells were counterselected on LB with 10% sucrose-containing medium with no added salt (3). Deletions were confirmed by colony PCR amplification and sequencing with primers flanking the gene. All sequencing was done at the Molecular Biology Core at the Geisel School of Medicine at Dartmouth.

### Construction of chromosomal point mutations.

Point mutations in the *pilY1* gene were made using a modified *in vitro* site-directed mutagenesis protocol (51). Forward and reverse complementary primers consisting of the nucleotide codon sequence encoding the mutation of interest were used to separately amplify the pMQ30 (for chromosomal mutations) and pMQ72 (ectopic expression) parental plasmids with the gene of interest using high-fidelity Phusion polymerase (NEB). After four cycles of amplification, the products of these reactions were combined and amplified for an additional 18 cycles with additional Phusion polymerase added. The parental plasmid was digested for 4 h using DpnI endonuclease (NEB) at 37°C. Following digestion, the PCR product was transformed into competent E. coli S17 cells and selected on LB with 10 μg/ml gentamicin. Plasmid containing the desired point mutation was isolated and confirmed by sequencing. Introduction of mutations on the chromosome was done by conjugation and counterselection as described above. All chromosomal mutations were verified by PCR amplification and sequencing.

### Rationale for construction of cysteine point mutations.

Before we identified the C152 residue as being important in separating the T4P in twitching versus signaling, based on the eukaryotic literature, we hypothesized that cysteines in PilY1 were important. However, we did not know which cysteines in PilY1 were important. The goal was to identify a cysteine point mutation that still twitched but lost signaling. We initially took a crude approach by mutating all the cysteines and then mutating all the cysteines except C300. Because sequence alignments indicated that C300 was highly conserved, we reasoned that C300 might be important in maintaining the structural integrity of the protein; i.e., if we mutated C300, the protein would most likely become unstable. Given the lack of twitching motility observed with these strains, they were of less interest. We therefore proceeded by making individual and double point cysteine mutations, hence the C232S/C241S mutants. Due to complications with cloning the C241S variant and given the genomic analysis and the subsequent discovery of the C152 residue’s role in signaling, we did not deem it worthwhile to build all possible single and double point mutations.

### Biofilm assay.

Overnight cultures (1.5 μl) were inoculated in U-bottom 96-well plates (Costar) containing 100 μl M8 salts minimal medium (Na_2_HPO_4_, KH_2_PO_4_, NaCl) supplemented with glucose (0.2%, vol/vol), Casamino Acids (0.5%, vol/vol), and MgSO_4_ (1 mM), subsequently referred to as M8 medium. Biofilm assay plates were then stained with 100 μl of 0.1% crystal violet in water for 20 min at room temperature and destained for 20 min with 125 μl destaining solution (40% glacial acetic, 40% methanol, and 20% [vol/vol] H_2_O). Absorbance was read at an optical density at 550 nm (OD_550_), and destaining solution was included as the blank. Biofilm assays were done similarly to those in published work by the O’Toole group (52, 53).

### *In vivo* c-di-GMP quantification.

Nucleotides were extracted from P. aeruginosa cells scraped from 0.52% agar with M8 medium after incubation for 37°C for 14 h. Cells were removed from plates by gently scraping the plate with a cover slip to avoid scraping the agar and then immediately placed on ice. Cell pellets were resuspended in 250 μl nucleotide extraction buffer (methanol-acetonitrile-distilled H_2_O [40:40:20] plus 0.1 N formic acid) and incubated at −20°C for 1 h. Following nucleotide extraction, cells were spun for 5 min at 4°C, and 200 μl of the supernatant was removed and then added to 8 μl of 15% NH_4_HCO_3_ stop solution. Nucleotides were dried in a speed vacuum and resuspended in 200 μl high-performance liquid chromatography (HPLC)-grade water (JT Baker) and placed in screw cap vials (Agilent Technologies). Quantification of c-di-GMP levels was done by liquid chromatography-tandem mass spectrometry (LC-MS/MS) by Lijun Chen at the Mass Spectrometry Facility at Michigan State University. All samples were normalized to dry weight and are expressed as picomoles divided by milligrams (dry weight).

### Macroscopic twitch assay.

One percent LB agar plates were stab inoculated to the bottom of the plate using toothpicks dipped in overnight cultures. Plates were incubated at 37°C for 24 h and for an additional day at room temperature. The agar was subsequently removed from the petri plate, the twitch zones were stained with crystal violet to visualize the zones, and the diameters of the twitch zones were measured.

### Plaquing assay.

One percent agar plates (60 × 15 mm) with M8 medium were prepared and cooled to room temperature. Fifty microliters of P. aeruginosa overnight culture was added to 1 mL of 0.5% warm top agar made with M8 medium and gently mixed. The mixture was quickly poured onto 1% agar plates made with M8 salts. Plates were swirled to ensure even spreading of the top agar. Once cooled, 2 μl of the phage DMS3*_vir_* strain was spotted at the center of the plate and allowed to dry, and the plate was subsequently incubated at 37°C overnight.

### Cell surface pili.

WT, Δ*pilY1* variant, *vWA* variant, and vWA-Cys152S cells were streaked in a grid-like pattern on 1% agar plates with M8 salts/supplements and incubated at 37°C overnight. Four plates per strain were struck for each biological replicate to ensure that an adequate number of pili could be recovered. The following day, cells were scraped off the plate using a cover slip, put in a 2-mL tube, and vortexed vigorously for 2 min with 1 mL of phosphate-buffered saline (PBS; Corning). Cells suspensions were subsequently spun at 16,000 × *g* for 5 min in a tabletop centrifuge, and the supernatant was removed and transferred to a clean tube and spun again. This step was repeated until no pelleted cells were recovered. Proteins were precipitated with 20% trichloroacetic acid (TCA; VWR) on ice overnight at 4°C. Precipitated proteins were collected by centrifuging cells at 16,000 × *g* for 25 min at 4°C. The supernatant was discarded, and the tubes were recentrifuged for 3 min to get rid of any remaining supernatant. Pellets were washed twice with 1 mL acetone (VWR) and subsequently air dried to remove residual acetone. Pellets were resuspended in 100 ml 1× sample buffer (Bio-Rad) with β-mercaptoethanol, boiled for 5 min, and then briefly spun before being run on a 12% SDS-PAGE gel, and the samples were probed for PilA and FliC by Western blotting. FliC served as the loading control and was used for the normalization of PilA protein levels. Samples were also resolved on a 7.5% SDS-PAGE gel and probed for PilY1 using a PilY1 antibody generously provided by Matt Wolfgang. Western blot analysis was performed as described below.

### Western blot analysis for PilY1 protein levels in WCL.

All strains were grown overnight in LB at 37°C. For whole-cell lysate (WCL) preparations, overnight cultures were diluted 1:100 in 5 mL M8 salt/supplement minimal medium and subcultured for ∼3 h at 37°C. Samples were resolved on a 7.5% Tris-HCl precast SDS-PAGE gel (Bio-Rad) and blotted onto a 0.45-μm-pore-size nitrocellulose membrane (Bio-Rad) using the 1.5-mm preprogrammed method on a Trans-Blot Turbo transfer system (Bio-Rad). The membrane was incubated in blocking buffer (LI-COR blocking buffer in Tris-buffered saline [TBS]) for 1 h at room temperature and incubated for 1 h or overnight at 4°C in polyclonal anti-PilY1 antibody (1:5,000 dilution) in bovine serum albumin (BSA)-TBS with Tween 20 (TBST [0.1%]) buffer. Following incubation with primary antibody, the membrane was washed in TBST (0.1%) for 5 min (three times) and incubated for 1 h with goat anti-rabbit antibody in TBST (0.1%) (1:10,000 dilution) secondary antibody (LI-COR IRDye 800CW goat anti-rabbit). Incubation with secondary antibody and all subsequent steps were performed in the dark. After incubation with the secondary antibody, the membrane was washed in TBST (0.1%) for 5 min (two times) and then once in TBS. The membrane was imaged using the LI-COR Odyssey CLx imager at the BioMT Core at the Geisel School of Medicine at Dartmouth. PilY1 protein levels were quantified relative to the cross-reacting band at ∼60 kDa using the LI-COR Image Studio Lite software by drawing a rectangle of the same size around each band and using the following background settings: average, a border width of 3, and the segment set at “all.”

### Protein quantification.

Total protein levels in whole-cell lysate were quantified using the Bio-Rad protein assay dye reagent per the manufacturer’s instructions as outlined by Bradford (54).

### AFM force spectroscopy.

Overnight cultures used in AFM experiments were diluted 200-fold in M8 salts, seeded on hydrophobic nontreated polystyrene petri dishes (Corning), and left for 10 min to adhere (26). Dishes were then washed gently but thoroughly with M8 salts medium to remove most nonadhered bacteria and used for AFM experiments in the same medium. AFM experiments were performed at room temperature using a NanoWizard 4 NanoScience AFM (JPK Instruments). Gold cantilevers (PNP-TR probes [Pyrex nitride probe with triangular cantilevers] from NanoWorld) were treated for 16 h with a 1 mM 1-dodecanethiol solution in ethanol to render them hydrophobic and then rinsed with ethanol and kept in MilliQ water until the AFM experiments were ready to be performed. Prior any measurements, the cantilever’s spring constant was empirically determined by the thermal noise method (55). The AFM force volume mode was used to record the force-distance curve in a pixel-by-pixel manner (force mapping) on 6- by 6-μm^2^ areas (32 by 32 pixels, i.e., 1,024 curves) with a bacterium at the center, previously localized by an optical microscope coupled to the AFM. For the Δ*pilA* strains overexpressing WT PilY1 or PilY1-Cys152S and lacking the pilus fiber, the area was decreased to 1 μm^2^ around the bacterium’s poles. The following settings were used: an applied force of 250 pN, a constant approach/retract speed of 5 μm/s, and a z-range of 1.5 μm.

### AFM data analysis.

Data were analyzed with the data processing software from JPK Instruments (Berlin, Germany). In a first approach, all force-distance curves exhibiting an adhesive event were selected, as opposed to the nonadhesive curves, which were discarded, thus allowing an estimation of the binding probability. In a second approach, adhesive curves were sorted depending on their signature (plateaus versus spikes), and the maximum adhesion sustained by each adhesive peak was determined. The frequency of plateaus was assessed by dividing the number of curves showing plateaus plus curves showing both plateaus and spikes by the total number of adhesive curves. A similar approach was used to calculate the percentage of spikes. The formulas to calculate the percent plateaus (*P*_plateaus_) or the percent spikes (*P*_spikes_) are shown below.

(1) $$P_{\text{plateaus}}=\left(N_{\text{curves showing only plateaus}}+N_{\text{curves showing plateaus and spikes}}\right)/\left(N_{\text{curves showing only plateaus}}+N_{\text{curves showing plateaus and spikes}}+N_{\text{curves showing only spikes}}\right)$$

(2) $$P_{\text{spikes}}=\left(N_{\text{curves showing only spikes}}+N_{\text{curves showing plateaus and spikes}}\right)/\left(N_{\text{curves showing only plateaus}}+N_{\text{curves showing plateaus and spikes}}+N_{\text{curves showing only spikes}}\right)$$

Statistical analyses were performed with Origin.

### Analysis of the IPCD database: generation of a phylogenetic tree, alignment, and calculation of Shannon diversity.

We performed nucleotide BLAST searches on a local version of the IPCD database of P. aeruginosa genomes to identify variants of the PilY1 protein. Using the nucleotide sequences of PilY1 from PA14 (GCF_000014625.1), PAO1 (GCF_000006765.1), and IPCD-83 (GenBank accession no. MCMY00000000), we were able to identify 852 strains with versions of the full protein. We used custom MATLAB scripts to perform an alignment of the amino acid sequences of all 852 versions of PilY1 and construct the corresponding phylogenetic tree. We performed the alignment of PilY1 amino acid sequences using a series of BLOSUM80 to BLOSUM30 scoring matrices. We constructed the phylogenetic tree of PilY1 sequences using a Jukes-Cantor maximum-likelihood method to estimate the number of substitutions between two sequences and an unweighted pair group method average (UPGMA) to construct the phylogenetic tree from the pairwise distances. One hundred twenty-nine sequences of PilY1 belong to a clade with highly similar proteins, which includes PilY1 from PA14. Seven hundred twenty-three sequences belong to a diverse clade that includes PilY1 from PAO1 and IPCD-83. Within each of these two groups, we calculated the Shannon diversity in each position along the aligned amino acid sequence using the formula $H=-\sum p_{i}\text{ln}(p_{i})$, where $p_{i}$ is the probability of each amino acid (including gaps). Code is available at https://github.com/GeiselBiofilm/IPCD-analysis.

### Growth assays.

Overnight cultures were inoculated in M8 salts/supplements at a starting OD_600_ of ∼0.05. Readings were taken every 40 min for 16 h in a Synergy Neo2-multimode microplate reader at the BioMT Core at the Geisel School of Medicine at Dartmouth.

### Cloning and protein expression of GST-vWA domain fusions.

The coding region of the WT and the C152S mutant of the vWA domain (amino acids 30 to 369) from P. aeruginosa PA14-UCBPP were PCR amplified and cloned into the pGEX-6p-1 plasmid at the BamHI cut site by Gibson assembly. Competent E. coli BL21(DE3) cells were transformed with the plasmid and selected on LB plates with 50 μg/mL carbenicillin grown at 30°C overnight. A single colony was used to inoculate 5 mL of liquid LB and grown for 12 to 14 h at 30°C. Each 5-mL seed culture was used to inoculate 500 mL LB in a 2-L flask and allowed to grow at 37°C with shaking at 200 rpm until the OD_600_ was 0.6 to 0.8. A total of 12 6-L LB flasks were inoculated. Expression was induced with 0.1 mM isopropyl-β-d-1-thiogalactopyranoside (IPTG) for 4 h at 37°C. Bacteria were harvested at 5,000 × *g* for 10 min, washed with PBS buffer, and stored at −20°C until further use.

### Purification of wild-type GST-vWA domain and Cys152S mutant proteins.

E. coli cells expressing WT GST-vWA domain and GST–vWA-C152S mutant proteins were resuspended in PBS supplemented with 2 mM TCEP [Tris(2-carboxyethyl)phosphine hydrochloride; Thermo Scientific], 0.01 mg/mL lysozyme from chicken egg (Sigma), EDTA-free protease inhibitor cocktail (Bimake), and 10 U/mL Benzonase nuclease (Millipore) and lysed in a Microfluidizer LM10 (Microfluidics) at 18,000 lb/in^2^. Nucleic acids were precipitated by addition of 0.1% polyethylenimine (branched; Sigma). Crude cell lysates were cleared by centrifugation at 200,000 × *g* for 1 h at 4°C in a Beckman Optima L-70 ultracentrifuge. Clear lysates were incubated overnight with 5 mL glutathione-Sepharose 4B resin (Cytiva) previously equilibrated with PBS containing 2 mM TCEP. Lysates and resin were transferred to a disposable plastic column and allowed to drain fully (flow through). Resin was washed with at least 15 column volumes of PBS–2 mM TCEP buffer before elution of the GST-vWA domain proteins with 5 column volumes of freshly prepared elution buffer (50 mM Tris-HCl, pH 8, 10 mM reduced glutathione). Elution fractions were concentrated using 30,000 molecular-weight-cutoff (MWCO), 15-ml Amicon centrifugal filters (Millipore) in a Beckman Allegra 6R centrifuge. Proteins were loaded in a HiLoad Superdex (75 pg; Cytiva) prepacked column equilibrated with 50 mM Tris-HCl (pH 8), 150 mM NaCl, and 1 mM TCEP using an AKTApure instrument (Cytiva). Fractions containing the fusion GST-vWA domain protein were combined and concentrated as described before and subjected to a second gel filtration step using a high-resolution Superdex 200 increase (10/300; Cytiva). Purified WT and C152S mutant GST-vWA domain proteins were extensively dialyzed against 20 mM sodium phosphate (pH 7.4) buffer. Final protein concentrations were determined using Bio-Rad protein assay reagent.

### CD and melting curves.

The far-UV circular dichroism (CD) spectra (195 to 250 nm) were recorded with a JASCO J-815 spectrophotometer (Jasco, Inc.) equipped with a CDF426S/15 Peltier temperature controller using a 2-mm path length quartz cuvette. CD spectra of proteins were recorded at 20°C using a step size of 0.1 mm. A time constant of 12 s was used to improve the signal-to-noise ratio and to decrease the contribution of the solvent at lower wavelengths. CD spectra were recorded using 1 μM GST-WT vWA domain and GST–vWA-C152S proteins in 20 mM sodium phosphate buffer, pH 7.4, and corrected by subtracting the spectrum of the buffer alone.

Thermal unfolding curves were obtained by monitoring the ellipticity at 222 and 208 nm of both fusion proteins at a 1 μM concentration at a heating rate of 1°C min^−1^ in the temperature range of 20 to 90°C. A 1-s integration time and 5-s equilibration time were used for each measurement, and buffer ellipticities at the selected wavelengths were subtracted from the samples data. Raw CD data were converted into the molar ellipticity [θ]_λ_ (degrees per square centimeter per decamole) at each wavelength using the relation [θ]_λ_ = θ_λ_/(10*CN*l), where θ_λ_ is the observed ellipticity in millidegrees at wavelength λ, *C* is the molar protein concentration, *N* is the number of amino acids of the protein, and l is the path length of the cuvette in centimeters. Following CD measurements, protein samples were collected and protein concentrations measured for accuracy.
