# Supplementary material for: Force-Induced Changes of PilY1 Drive Surface Sensing by Pseudomonas aeruginosa
Source: mBio. 2022 Feb 1;13(1):e03754-21. doi: 10.1128/mbio.03754-21 (PMC8806160; doi:10.1128/mbio.03754-21)
Supplement: FIG S1 [file mbio.03754-21-sf001.pdf]

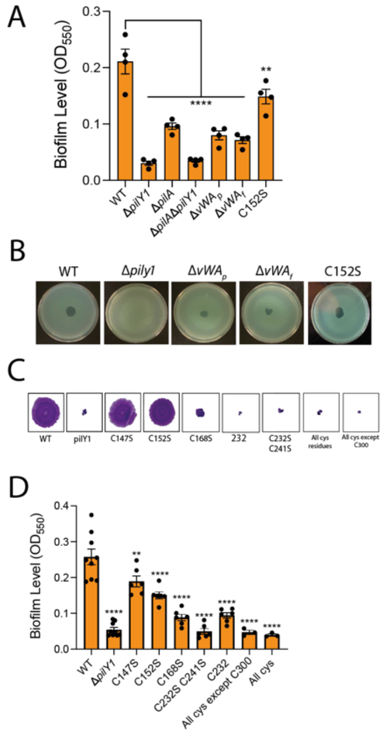

Figure S1. A. Biofilm formation measured at OD_{550} for WT, Δ*pilY1*, Δ*pilA,* Δ*pilA* Δ*pilY1,* the vWA variants, and the Cys152S mutant in a static 96 well biofilm assay performed in M8 medium salts plus supplements incubated at 37 °C for 24 h. The Δ*pilA* strain shows a less severe phenotype than the Δ*pilY1* strain indicating that PilY1 has functions independent of its impact on T4P biogenesis, as previously described [3]. **B-C.** Partial functionality of vWA variants and phenotypic analysis of other cysteine vWA mutants. **B.** Plaquing assay with phage $DMS3_{vir}$ versus the WT and the indicated mutants as hosts. Zones of clearing shown for WT and the strain expressing the vWA-Cys152S mutant protein are similar, which indicates a similar degree of TFP function. The Δ*pilY1* mutant serves as the negative control. **C.** Representative images of twitch zones stained with crystal violet shown for WT, the Δ*pilY1* or strains expressing PilY1 variants with point mutations in the Cys residues in the vWA domain following incubation at 37 °C for 24 h plus one additional day at room temperature. Twitching serves as a measure of TFP function. **D.** Biofilm level measured at OD_{550} for WT and the mutants shown in panel B using the 96 well static biofilm assay after 24 hrs at 37 °C, as described in the Materials and Methods.
