# Supplementary material for: Force-Induced Changes of PilY1 Drive Surface Sensing by Pseudomonas aeruginosa
Source: mBio. 2022 Feb 1;13(1):e03754-21. doi: 10.1128/mbio.03754-21 (PMC8806160; doi:10.1128/mbio.03754-21)
Supplement: FIG S2 [file mbio.03754-21-sf002.pdf]

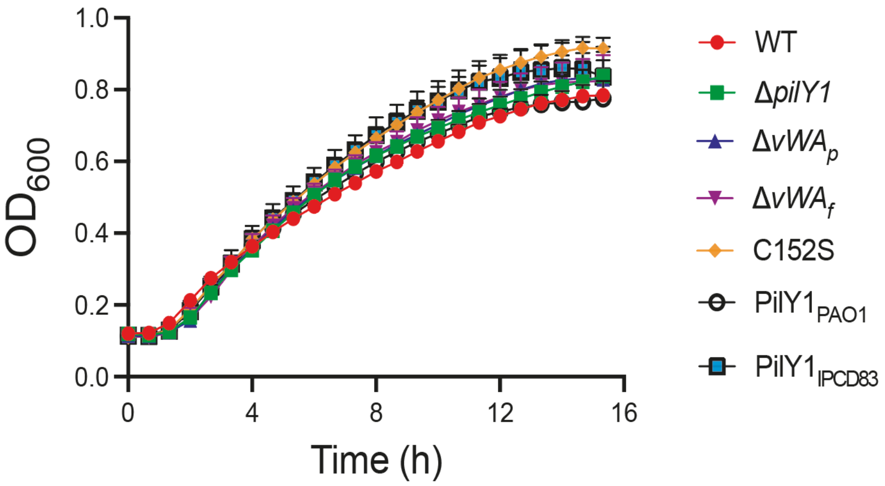

Figure S2. Growth curves for WT and the strains expressing the PilY1 variants. Growth assays were performed in M8 minimal salts medium supplemented with casamino acids, glucose and magnesium sulfate. This medium was also used for all macroscopic biofilm assays, c-di-GMP measurements, plaquing assays and AFM studies. The data are from three biological replicates each with two technical replicates. There is no significant difference among the growth kinetics of each strain. Error bars show SEM and statistical significance was determined at each time point using one-way analysis of variance (ANOVA) using multiple comparisons test.
