# Supplementary material for: Force-Induced Changes of PilY1 Drive Surface Sensing by Pseudomonas aeruginosa
Source: mBio. 2022 Feb 1;13(1):e03754-21. doi: 10.1128/mbio.03754-21 (PMC8806160; doi:10.1128/mbio.03754-21)
Supplement: TABLE S1 [file mbio.03754-21-st001.pdf]

Table S1. Strains used in this study

SMC #s/Strain Name	Genotype/Description	Source
***E. coli* strains**		
S17-1 λ pir	*thi pro hsdR- hsdM+ ΔrecA RP4-2::TcMu-Km::Tn*	
BL21-DE3	Protein expression strain	NEB

***P. aeruginosa* strains**

232	PA14-UCBPP wildtype (WT)	[1]
8929	WT mKate pPcdrA::GFP	[2]
	C152S pPcdrA::GFP	This study
3718	Δ*pilY1*	[3]
7445	ΔvWA_p (amino acids S178-S365)	This study
9142	ΔvWA_f	This study
9144	PilY1-C147S	This study
9146	PilY1-C152S	This study
9148	PilY1-C168S	This study
9150	PilY1-C232S	
9152	PilY1-C232S C241S	This study
9154	PilY1-all seven cysteines in vWA mutated to serine	This study
9156	PilY1-all cysteines in vWA mutated to serine except C300	This study
9158	PilY1 from WT PAO1 swapped in for PA14's PilY1	This study
9160	PilY1 from IPCD83 swapped in for PA14's PilY1	This study
9082	Δ*pilA*	[4]
9135	Δ*pilA* empty vector	This study
9119	Δ*pilA* pPilY1-His	This study
9120	Δ*pilA* pPilY1 ΔvWA_f-His	This study
9121	Δ*pilA* pPilY1-C152S-His	This study

References

1. Rahme, L.G., et al., *Common virulence factors for bacterial pathogenicity in plants and animals.* Science, 1995. **268**(5219): p. 1899-902.
2. Webster, S.S., et al., *Interaction between the type 4 pili machinery and a diguanylate cyclase fine-tune c-di-GMP levels during early biofilm formation.* Proc Natl Acad Sci U S A, 2021. **118**(26).
3. Bohn, Y.S., et al., *Multiple roles of Pseudomonas aeruginosa TBCF10839 PilY1 in motility, transport and infection.* Mol Microbiol, 2009. **71**(3): p. 730-47.
4. Luo, Y., et al., *A hierarchical cascade of second messengers regulates Pseudomonas aeruginosa surface behaviors.* mBio, 2015. **6**(1).
