# Supplementary material for: Force-Induced Changes of PilY1 Drive Surface Sensing by Pseudomonas aeruginosa
Source: mBio. 2022 Feb 1;13(1):e03754-21. doi: 10.1128/mbio.03754-21 (PMC8806160; doi:10.1128/mbio.03754-21)
Supplement: TABLE S2 [file mbio.03754-21-st002.pdf]

Table S2. Plasmids used in this study

Plasmid	Description	Source/SMC #
pMQ30	Shuttle vector for yeast cloning and Gram-negative allelic replacement, Gm^r	
	vWA_p KI construct, Gm^r	This study/7254
	vWA_f KI construct, Gm^r	This study/9141
	PilY1-C147S KI construct, Gm^r	This study/9143
	PilY1-C152S KI construct, Gm^r	This study/9145
	PilY1-C168S KI construct, Gm^r	This study/9146
	PilY1-C232S KI construct, Gm^r	This study/9148
	PilY1-C232S C241S KI construct, Gm^r	This study/9150
	PilY1-all cysteines mutated to serine KI construct, Gm^r	This study/9152
	PilY1-all cysteines mutated to serine except C300 KI construct, Gm^r	This study/9154
	PAO1's pilY1 KI (to KI to PA14)	This study/9156
	IPCD83's pilY1 KI (to KI to PA14)	This study/9158
pMQ72	expression vector with arabinose-inducible gene expression; Gm^r	[1]
	pPilY1-His	This study/9171
	pPilY1 ΔvWA_f-His	This study/9172
	pPilY1-C152S-His	This study/9173
pGEX-GST expression plasmid	*vWA_f-His	This study/9126
	*vWA_f-C152S-His	This study/9136

*Sequence codon optimized for E. coli
KI – knock in

1. Shanks, R.M., et al., Saccharomyces cerevisiae-based molecular tool kit for manipulation of genes from gram-negative bacteria. Appl Environ Microbiol, 2006. 72(7):5027-36.
