# Supplementary material for: Force-Induced Changes of PilY1 Drive Surface Sensing by Pseudomonas aeruginosa
Source: mBio. 2022 Feb 1;13(1):e03754-21. doi: 10.1128/mbio.03754-21 (PMC8806160; doi:10.1128/mbio.03754-21)
Supplement: TABLE S3 [file mbio.03754-21-st003.pdf]

Table S3. Oligonucleotides used in this study

Primer Name	Primer Sequence (5' – 3')	Purpose
QC vWA C147S-F	cgccctcaatggggaaccggctggcttggtagcatcgatagcagctgcaataccgggag	Site-directed mutagenesis
QC vWA C147S-R	ctcccggtattgcagctgctatcgatgctaccaagccagccggttccccattgagggcg	Site-directed mutagenesis
QC vWA C152S-F	ccggctggcttggttgcatcgatagcagcagcaataccgggagagcttattactatac	Site-directed mutagenesis
QC vWA C152S-R	Gtatagtaataagctctcccggtattgctgctgctatcgatgcaaccaagccagccgg	Site-directed mutagenesis
QC vWA C168S-F	ctatacttataaggtaagcgctagcagccctgcacagccggtgagcagctccaactcc	Site-directed mutagenesis
QC vWA C168S-R	ggagttggagctgctcaccggctgtgcagggctgctagcgcttaccttataagtatag	Site-directed mutagenesis
QC PilY1 C232S-F	gtctcacttgggggccctgaacaccagtagcatcggcgccaacagcagaagctgcc	Site-directed mutagenesis
QC PilY1 C232S-R	ggcagcttctgctgttggcgccgatgctactggtgttcagggcccccaagtgagac	Site-directed mutagenesis
QC PilY1 S300C-F	ccaccgaagacggaaagacatattcctgccgggccagctatcacatcatgatgac	Site-directed mutagenesis
QC PilY1 S300C-R	gtcatcatgatgtgatagctggcccggcaggaatatgtctttccgtcttcggtgg	Site-directed mutagenesis
QC PilY1C300S F	ccaccgaagacggaaagacatattccagccgggccagctatcacatcatgatgaccgac	Site-directed mutagenesis
QC PilY1 C300S R	gtcggtcatcatgatgtgatagctggcccggctggaatatgtctttccgtcttcggtgg	Site-directed mutagenesis
QC PilY1_C232/241S F	gggccctgaacaccagcagcatcggcgccaacagcagaagcagccaaaacaatgccctg	Site-directed mutagenesis
QC PilY1_C232/241S_R	cagggcattgttttggctgcttctgctgttggcgccgatgctgctggtgttcagggccc	Site-directed mutagenesis
QC vWA-His C152S F pGEX	ggttggcttggatgcatcgattcctcatccaatactggccgcgcttactactatac	For making point mutation in pGEX plasmid
QC vWA-His C152S R pGEX	gtatagtagtaagcgcggccagtattggatgaggaatcgatgcatccaagccaacc	For making point mutation in pGEX plasmid
vWA5A KI-F	ccagtgccaagcttgcatgcctgcaggtcgactttccaacatgcgcgaggtgtcactg	For making eukaryotic vWA KI in pMQ30

vWA5A KI-R	attacgaattcgagctcggtacccggggatcctggagctcggttgtccagcagtgcgcc	For making eukaryotic vWA KI in pMQ30
IPCD83 F	gtataccaactgactggagccagcgcATGAAATCGGCACTCCACCAGATCGGCAAG	Forward primer with homology to *pilY1* from IPCD83. Highlighted region is homologous to region upstream of PA14's *pilY1.*
IPCD83 R	gagctgtggcgagaagacgtaaggggttcatgtTCAGTTCTTTCCTTCGATGGGGCGC	Reverse primer with homology to *pilY1* from IPCD83. Highlighted region is homologous to region downstream of PA14's *pilY1.*
pilY1-KI F	tgtaaaacgacggccagtgccaagcttgcatGCCTGCCATACCTCTCGACAGCAGGGAT	Forward primer with homology to *pilY1* to pmQ30 (red).
pilY1-KI R	ccatgattacgaattcgagctcggtacccggggATCCGATAACCTCCGTCGTTGGCTAC	Reverse primer with homology to *pilY1* to pmQ30 (red).
PAO1 PilY1 PA14 up F	gtgccaagcttgcatgcctgcaggtcgactTTTTCAATACCATCAAGCCCCCAGAGGTC	Forward primer to amplify upstream region of PA14's PilY1. Region in red highlight homology to pmQ30 plasmid
PAO1 PilY1 PA14 up R	tcttgccgatctggtggagtaccgatttcatgCGCTGGCTCCAGTCAGTTGGTATACAG	Forward primer to amplify upstream region of PA14's PilY1. Region in red highlight homology to pmQ30 plasmid
PilY1 PAO1 gene 14 tail F	ctgtataccaactgactggagccagcgcATGAAATCGGTACTCCACCAGATCGGCAAGA	Forward primer to amplify PAO1's PilY1. Region in red highlight homology upstream of PA14's PilY1
PilY1 PAO1 gene 14 tail R	gcgagaagacgtaaggggttcatgtTCAGTTCTTTCCTTCGATGGGGCGCCAGTTC	Reverse primer to amplify PAO1's PilY1. Region in red highlight homology downstream of PA14's PilY1
PilY1 PAO1 downstream-F	gaactggcgccccatcgaaggaaagaactgaACATGAACCCCTTACGTCTTCTCGC	Forward primer to amplify downstream region of PA14's PilY1. Region in red highlight homology to PAO1's PilY1 plasmid
PilY1 PAO1 downstream-R	attacgaattcgagctcggtacccggggatcctGTGAAAAATAGCGCTCTTGAGTAGC	Reverse primer to amplify downstream region of PA14's PilY1. Region in red

		highlight homology to PAO1's PilY1 plasmid
pPilY1-His_pmq72 F	ctagcgaattcgagctcggtacgaaggagatatacatatgatccaccagattac ccgcg	Forward primer to amplify pilY1 and insert in pmQ72 multi-copy plasmid. Regions in green and red represent the RBS and homology to the plasmid, respectively.
pPilY1-His pmq72 R kpn1	gaggatccccgggtacTCAgtggtgatggtggtggtgtttctcctcgacgaccc gccag	Reverse primer to amplify pilY1 and insert in pmQ72 multi-copy plasmid. Regions in blue and red are the His tag and homology to the plasmid, respectively.
